# Factors affecting the buccal delivery of deformable nanovesicles based on insulin–phospholipid complex: an *in vivo* investigation

**DOI:** 10.1080/10717544.2020.1778814

**Published:** 2020-06-29

**Authors:** Yuqi Yang, Yiyue Guo, You Xu, Yingying Meng, Xing Zhang, Xuejun Xia, YuLing Liu

**Affiliations:** State Key Laboratory of Bioactive Substance and Function of Natural Medicines, Beijing Key Laboratory of Drug Delivery Technology and Novel Formulations, Department of Pharmaceutics, Institute of Materia Medica, Chinese Academy of Medical Sciences & Peking Union Medical College, Beijing, PR China

**Keywords:** Buccal delivery, in vivo, deformable nanovesicles, insulin–phospholipid complex, drug concentration, size, deformability

## Abstract

Deformable nanovesicles (DNVs) have been used in the buccal delivery of biomacromolecules due to their ability to enhance drug penetration. However, no breakthroughs have been made until now due to limited understanding of the factors affecting *in vivo* buccal delivery. In this study, we designed a series of DNVs, based on an insulin–phospholipid complex (IPC-DNVs), to investigate the influence of drug dose, buccal administration methods, and key quality characteristics of IPC-DNVs for buccal delivery. IPC-DNVs showed a non-linear dose–response relationship between 8 and 12 IU. There was no significant effect of drug delivery site (sublingual mucosa/buccal mucosa) or ligation time (15 or 30 min) on buccal absorption of IPC-DNVs. However, the area above the curve of reduction in blood glucose level overtime (AAC_0–6h_) for oral mucosa administration was significantly higher than that for buccal mucosa administration. Increasing the drug concentration in IPC-DNVs led to a decrease in AAC_0–6h_. This might be due to local leakage of DNVs, while squeezing through biological barriers with high concentration of insulin, thus hindering the subsequent delivery of DNVs. IPC-DNVs, measuring 80–220 nm in size, did not significantly affect AAC_0–6h_. However, when the size was increased to approximately 400 nm, AAC_0–6h_ decreased, thus suggesting that IPC-DNVs with reasonable size were more effective. Additionally, increased deformability of IPC-DNVs might cause drugs to leak easily, thus reducing the promoting effect of buccal absorption. Our results clarified the effect of characteristics of IPC-DNVs on buccal delivery *in vivo* and provided meaningful support for the design of dosage form of DNVs.

## 1. Introduction

With continuing advances in biotechnology and genetic engineering, there has been a dramatic increase in the availability of new biomacromolecules, which have the potential to ameliorate the symptoms of many poorly treated diseases. However, most biological macromolecules, such as proteins and peptides, must be administered by injection due to physio-chemical and biological restrictions, often leading to poor patient compliance. Hence, the need to realize alternative noninvasive routes to administer biomacromolecules has always been an important issue in the field of drug research.

Among the different alternative noninvasive routes of drug delivery, such as buccal delivery, transdermal delivery (Herwadkar & Banga, 2012), intranasal delivery (Meredith et al., 2015), and pulmonary delivery (Adjei & Gupta, 1994), the buccal route has garnered maximum attention since the buccal mucosa is visible, resilient, protected by saliva, and highly vascular (Patel et al., 2011). However, there are some intrinsic limitations related to buccal administration of drugs. One major challenge is the poor permeability of an organized array of neutral lipids through the superficial layers of the epithelium (Senel et al., 2001). Currently, physical or chemical enhancement techniques are used to assist the biomacromolecules to cross the mucosa. Deformable nanovesicles (DNVs), which can be squeezed through biological barriers that are much smaller than its own diameter without physical assistance (Cevc & Blume, 1992), have proven to be a potential drug delivery carrier which can effectively cross a variety of biological barriers. However, due to the specific characteristics of the oral environment and DNVs, the actual drug delivery efficiency of DNVs in oral cavity is influenced by several factors, including the quality of DNVs and drug administration methods. No breakthrough has been made so far due to our limited understanding of the factors affecting buccal delivery of DNVs *in vivo*. It is crucial to bridge this gap to optimize the formulation of DNVs and decrease the risk of failure in clinical trials.

The drug delivery ability of DNVs is highly dependent on their quality. For example, although DNVs with increased deformability have increased drug delivery ability (Morilla & Romero, 2016), the drug leakage rate during the permeation process is also increased. Similarly, increasing the size of DNVs may not be conducive to drug delivery, since DNVs with a relatively large size may break up and produce fragments, resulting in drug leakage during permeation (Cevc, 1995). Fragments of DNVs and leaked drugs, such as insulin–phospholipid complexes (IPC), may have impact on mucosal permeability. Thus, it is difficult to predict the effect of the quality of DNVs on buccal delivery. Compared to other noninvasive drug delivery routes, buccal delivery is also influenced by the continuous secretion of saliva in the oral cavity which can alter the drug concentration in DNVs and drug release per unit time, thus affecting the final efficacy of buccal delivery. In addition, although scientists have clarified that sublingual mucosa has better permeability than buccal mucosa, the buccal mucosa has a larger surface area and abundant blood flow compared to sublingual mucosa (Patel et al., 2011). Thus, it is still not clear which mucosa provides better absorption of drugs. Therefore, understanding the relationship between the quality of DNVs, the method of administration, and efficacy of buccal delivery efficacy is of vital importance.

In our previous study, we developed a novel DNVs loaded with IPC (IPC-DNVs) for buccal absorption. These IPC-DNVs had a high drug entrapment efficiency and high relative pharmacological bioavailability (15.53%) (Xu et al., 2018). Additionally, freeze-drying technology was used to fabricate lyophilized IPC-DNVs, using lactose and trehalose (1:4) as the protective agents. The freeze-dried sample could be stored under 25 °C for more than 6 months and had a relative pharmacological bioavailability of 14.49% (Xu et al., 2019). In this study, we aimed to evaluate the buccal delivery characteristics of IPC-DNVs *in vivo* and provide support for dosage form design and administration methods for buccal delivery system of DNVs. We also examined the stability of IPC-DNVs when exposed to buccal enzymes. In addition, we designed a series of IPC-DNVs with different quality characteristics by adjusting drug concentration, size, or deformability based on previous IPC-DNVs, and investigated the factors affecting IPC-DNVs on buccal delivery *in vivo*, including drug dose, buccal administration method (delivery site, ligation time), and the key quality characteristics of IPC-DNVs. Normal rabbit esophageal ligation was used as the animal model to assess the hypoglycemic effect after buccal administration of IPC-DNVs. The ability of buccal delivery of IPC-DNVs was evaluated by calculating the area above the curve of reduction in blood glucose level over time (AAC_0–6h_) and the relative pharmacological bioavailability (Fp).

## 2. Materials and methods

### 2.1. Materials

Recombinant human insulin was purchased from Dongbao Enterprise Group Co. Ltd. (Jilin, People’s Republic of China). Lecithin (70% phosphatidylcholine/30% phosphatidylethanolamine, Lipoid S75) was obtained from Shanghai Toshisun Biology & Technology Co. Ltd. (Shanghai, People’s Republic of China). Sodium deoxycholate (SDC), D-(+)-Trehalose and sodium pentobarbital were obtained from Sigma-Aldrich (St. Louis, MO, USA). α-Lactose monohydrate was purchased from Aladdin (Shanghai, People’s Republic of China). Other chemicals and solvents used were of analytical or chromatographic grade.

### 2.2. Animals

Male big-eared Japanese rabbits were purchased from Beijing HFK Bioscience Co. Ltd. (Beijing, People’s Republic of China). All rabbits were raised in the Institute of Materia Medica, Chinese Academy of Medical Sciences & Peking Union Medical College (Beijing, People’s Republic of China). The experiments were performed with the approval of the Laboratory Animal Care and Use Committee of the Peking Union Medical College.

### 2.3. Preparation

#### 2.3.1. IPC-DNVs

IPC-DNVs were prepared using the thin-film hydration method, as described in our previous study (Xu et al., 2018). Briefly, a specified amount of lipid S 75 (600 mg) and insulin (60 mg) were taken in a round-bottom flask, and separately dissolved in dichloromethane (54 mL) and 0.1% trifluoroacetic acid-methanol (6 mL). After mixing, IPC was formed by rotary evaporation at 37 °C. Next, IPC was placed under vacuum suction overnight to remove the residual organic solvent. Appropriate amounts of IPC, Lipoid S75 (600 mg), and Tween 20 (400 mg) were dissolved in dichloromethane to form a clear solution, and the organic solvent was evaporated at 37 °C using a rotary evaporator to yield a thin lipid film. The resulting thin film was suspended in phosphate-buffered solution (PBS, pH 7.4), containing sodium deoxycholate (100 mg), by rotation at 150 rpm for 30 min at 37 °C. To prepare IPC-DNVs, the above suspension was sonicated four times (1 min each), under cooling, to yield small-sized IPC-DNVs. The obtained DNVs were further passed through 0.22 μm polycarbonate membranes for homogenization. The final concentrations of insulin and SDC were 3 mg/mL and 0.5%, respectively.

#### 2.3.2. IPC-DNVs with different deformabilities

The preparation method of IPC-DNVs with different deformabilities was the same as that of IPC-DNVs, except for changing the amount of SDC used (100 mg, 0.5%; 200 mg, 1%).

#### 2.3.3. IPC-DNVs with different drug concentrations and sizes

To obtain IPC-DNVs with different drug concentrations and sizes, lyophilized IPC-DNVs were prepared using the freeze-drying technology, as described in our previous study (Xu et al., 2019). Briefly, 8% cryoprotectant (lactose and trehalose in a 1:4 ratio) was dissolved in PBS, and 0.5 mL solution of IPC-DNVs was diluted to 1 mL with PBS. Lyophilized IPC-DNVs were obtained using the following freeze-drying procedure. The sample plate was first cooled to a temperature of −45 °C at a rate of 20 °C/h and maintained for 3 h. The plate temperature was then raised to −25 °C, at a rate of 5 °C/h and maintained for 10 h. The plate was then slowly heated to −10 °C for 3 h, heated to 0 °C for 3 h, and heated to 10 °C for 3 h, at a rate of 5 °C/h. Finally, the plate was returned to room temperature and left at the same temperature for 3 h.

Lyophilized IPC-DNVs were reconstituted by dispersing in distilled water to the original volume to form liquid IPC-DNVs, and the concentration of insulin was 3 mg/mL. IPC-DNVs with different drug concentrations (3 mg/mL, 4 mg/mL, 5 mg/mL) were prepared by dissolving lyophilized IPC-DNVs in different volumes of distilled water. IPC-DNVs with different sizes (110 nm, 160 nm, 220 nm, 400 nm) were prepared by changing the amount and proportion of the cryoprotectant (lactose and trehalose) in lyophilized IPC-DNVs.

### 2.4. Characterization

#### 2.4.1. Determination of size

The average particle size and size distribution were determined by dynamic light scattering technique, using a Zetasizer Nano ZSP (Malvern Instruments Ltd, Malvern, UK), at an angle of 173° at 25 °C. Prior to analysis, the samples were diluted ten times with distilled water.

#### 2.4.2. Entrapment efficiency

The entrapment efficiency of deformable nanovesicles based on insulin–phospholipid complex (IPC-DNVs) was determined by a fast ultrafiltration method, as described previously (Xu et al., 2018). Briefly, the concentration of insulin in IPC-DNVs was determined using high-pressure liquid chromatography (HPLC) with an Agilent Technologies 1200 series HPLC system (Agilent, Santa Clara, CA, USA), equipped with a 300SB-C18 column (4.6 × 250 mm, 5 μm, Agilent). The measuring conditions were as follows: 0.2 M sulfate buffer/acetonitrile (74:26, v/v), flow rate: 1 mL/min, ultraviolet detection: 214 nm, injection volume: 20 μL, and column oven temperature: 40 °C. Content determination was carried out by dissolving IPC-DNVs suspensions in PBS (0.02 mM, pH 7.4). First, 100 µL of DNVs suspension was diluted to 10 mL using PBS (0.02 mM, pH 7.4). Subsequently, the amount of insulin in the solvent was measured using HPLC.

A fast ultrafiltration method was used to determine the entrapment efficiency (EE%) of IPC-DNVs. Briefly, 1 mL of each prepared IPC-DNVs suspension was placed in a centrifugal filter tube (Amicon Ultra-4 centrifugal devices, 100 K NMWL; Millipore, Billerica, MA, USA) and centrifuged at 4000 rpm for 40 min to separate free and entrapped insulin in the IPC-DNVs. After ultrafiltration, the amount of insulin in the ultrafiltrates was measured using RP-HPLC. Subsequently, the centrifugal filter tube was washed thrice with PBS at 2500 rpm for 10 min and the ultrafiltrate was collected and analyzed using the RP-HPLC to determine the drug absorbed by the centrifugal filter tube. EE% was calculated according to the following equation:

$$EE\%=\frac{W_{\text{total}-\mathrm{insulin}}-(W_{\mathrm{free}-\mathrm{insulin}}+W_{\mathrm{absorbed}-\mathrm{insulin}})}{W_{\mathrm{total}-\mathrm{insulin}}}$$

where W_total-insulin_ is the total amount of insulin in DNVs suspension, W_free-insulin_ is the amount of insulin in the ultrafiltrates, and W_absorbed-insulin_ is the absorbed insulin on the centrifugal filter. Each experiment was performed in triplicate.

#### 2.4.3. Deformability

The deformability of IPC-DNVs was determined using a stainless-steel pressure filter device. A constant pressure of 0.45 MPa was applied for 5 min to extrude the IPC-DNVs through the 50 nm polycarbonate membranes. The size of IPC-DNVs was measured using the size analyzer, as mentioned previously. The deformability index of the IPC-DNVs (DI, g/cm^2^/s) was calculated according to the following formula (Zeb et al., 2016).

$$DI=J*{\left(\frac{r_{v}^{}}{r_{p}}\right)}_{}^{2}$$

where *J* is the rate of penetration through the permeable membrane, r_v_ is the size after extrusion, and r_p_ is the pore size of the permeable membrane (50 nm).

### 2.5. Drug release *in vitro*

The drug release of IPC-DNVs *in vitro* was determined by dialysis bag method with phosphate buffer at pH 7.4 *in vitro*. Briefly, a 1 mL volume of IPC-DNVs was placed in the dialysis bag (16 mm, MW: 100KD) from Spectrum Labs (USA), and introduced into 15 mL of release media at 37 °C, with a rotation speed of 80 rpm. For the release studies, 15 mL of sample was taken out and replaced with 15 mL fresh medium at, 1, 3, 8 and 12 h. Each study was carried out in triplicate and the concentrations of insulin was determined using reverse phase high-performance liquid chromatography (RP-HPLC) with an Agilent Technologies 1200 series HPLC system (Agilent, Santa Clara, CA, USA) and a 300SB-C18 column (4.6 × 250 mm, 5 μm, Agilent). The drug release profile was compared with that of insulin solution.

### 2.6. Stability of IPC-DNVs against degradation by buccal enzymes

The stability of insulin solution and IPC-DNVs, against degradation by isolated aminopeptidase N was evaluated by incubating them with artificial saliva, containing isolated aminopeptidase N. Briefly, 100 µL of IPC-DNVs suspension was diluted to 10 mL with artificial saliva, containing aminopeptidase N (0.625 μg/mL), and incubated in a shaking water bath (100 rpm) at 37 °C. Aliquots (500 μL) were withdrawn at fixed intervals and put in an ice bath for 2 min to stop the reaction. Thereafter, the samples were analyzed using the RP-HPLC assay. Each experiment was performed in triplicate.

### 2.7. *In vivo* hypoglycemic effect of IPC-DNVs

The relative pharmacological bioactivity of insulin delivered via the transmucosal route by IPC-DNVs in different drug doses, delivery sites, ligation times, drug concentrations, sizes, and deformabilities was evaluated in normal rabbits by assessing the hypoglycemic effects.

Before the experiment, the rabbits were randomly divided into groups of three as follows:

- Insulin solution subcutaneous administration group (INS-subcutaneous): 3 mg/mL, 1 IU/kg.
- Insulin solution buccal administration control group (control): 3 mg/mL, 10 IU/kg.
- IPC-DNVs group (IPC-DNVs, 3 mg/mL): drug dose (8 IU/kg, 10 IU/kg, 12 IU/kg); delivery site (buccal mucosa, sublingual mucosa, oral mucosa), 10 IU/kg; ligation time (15 min, 30 min), 10 IU/kg; buccal administration.
- IPC-DNVs with different quality characteristics group: drug concentration (3 mg/mL, 4 mg/mL, 5 mg/mL) ; size (target size: 110 nm, 160 nm, 220 nm, 400 nm); deformability (0.5% SDC, 1% SDC); 10 IU/kg; buccal administration.

After fasting for 2 h, the rabbits were weighed and anesthetized with pentobarbital (2%, 50 mg/kg) through the ear margin vein. During anesthesia, the animals were restrained in a supine position to a board. The esophagus was surgically ligated to prevent swallowing. The drug solution for buccal administration (oral mucosa) was divided into four parts; two parts for administration via sublingual mucosa, and two parts for administration via left and right buccal mucosa. The sublingual mucosa and the buccal mucosa groups were administered only to the sublingual mucosa or buccal mucosa. After administration of the drug solution for 30 min (except the group with ligation time of 15 min), the esophagus was untied, and the response of the animals was observed. Blood samples were collected from the ear veins at predetermined time intervals (0, 0.5, 1, 1.5, 2, 2.5, 3, 3.5, 4, 4.5, 5, and 6 h) after DNVs administration and blood glucose levels were measured using a Blood Analyzer (ONETOUCH Ultra; Johnson & Johnson, New Brunswick, NJ, USA). Only the animals with initial blood glucose value between 7 and 10 mmol/L and those which maintained a constant body temperature throughout the experiment were selected for buccal administration. At the end of the experiment, the animals were euthanized by administering an intravenous overdose of pentobarbital (2%).

The relative pharmacological bioavailability (Fp) of insulin, after buccal administration, was calculated using the following equation (Lee et al., 2017):

$$\mathrm{Fp}=\frac{{\mathrm{AAC}}_{\mathrm{buccal}}\times {\mathrm{Dose}}_{s.c.}}{{\mathrm{AAC}}_{s.c.}\times {\mathrm{Dose}}_{\mathrm{bucc}al}}\times 100\%$$

where Dose_buccal_ and Dose_s.c._ are the insulin doses administered through the buccal or subcutaneous route, respectively, and AAC is the area above the curve of the reduction in blood glucose level over time.

## 3. Statistical analysis

The data were expressed as the mean ± standard deviation of three independent experiments and analyzed by one-way analysis of variance. Student’s *t*-test was performed, using SPSS software, to determine statistically significant differences between the groups. A *p*-value of less than .05 was considered to be statistically significant: *p < .05, **p < .01.

## 4. Results and discussion

### 4.1. Stability of IPC-DNVs against degradation by buccal enzymes

The stability of IPC-DNVs against degradation by buccal enzymes was assessed in this study, considering that previous studies did not assess the propensity of IPC-DNVs toward degradation by buccal enzymes. The activity of buccal cavity enzymes is relatively low compared to that of digestive enzymes. Nevertheless, buccal administration of some peptide drugs remains limited due to degradation by membrane-bound peptidases. Aminopeptidase N is the most abundant peptidase found on the surface of the buccal mucosa (Langoth et al., 2005). In this study, isolated aminopeptidase N was used to evaluate the stability of insulin and IPC-DNVs in the buccal cavity by determining whether pure insulin and IPC-DNVs were stable in artificial saliva, containing aminopeptidase N. The metabolism of insulin was monitored by measuring the decrease in peak area of native insulin over time. After 3 h of incubation with aminopeptidase N, at a concentration of 0.625 μg/mL, the remaining percentage of insulin in the insulin solution and IPC-DNVs was 95.86% and 98.84%, respectively. The degree of degradation in the two groups was not significantly different. The results indicate that both free insulin and insulin entrapped in the DNVs was stable after buccal administration, thus providing evidence for the buccal administration of insulin.

### 4.2. Characterization

In this study, a series of IPC-DNVs (Xu et al., 2018) and lyophilized IPC-DNVs (Xu et al., 2019), were prepared to study the factors that influence buccal drug delivery. In our initial design, we considered obtaining IPC-DNVs, with different drug concentrations and sizes, by changing the hydration volume and ultrasonic power or time, respectively. However, it was found that reduction in the hydration volume might lead to incomplete hydration. Nanovesicles of different sizes by altering ultrasonic power or time were also difficult to obtain. Therefore, we prepared IPC-DNVs with different drug concentrations and sizes based on lyophilized IPC-DNVs. As described in our previous studies, the particle sizes of IPC-DNVs or reconstituted lyophilized IPC-DNVs were 85.84 nm and 109.23 nm, respectively, and both had a narrow size distribution, obvious whorls structure, high EE%, and deformability.

Based on the above results, we designed a series of IPC-DNVs with different quality characteristics by adjusting the drug concentration, size, or deformability. The main quality indices were determined (Tables 4–6). The change in drug concentration had no effect on the size, distribution, and EE% of DNVs. Changing the amount of cryoprotectant and proportion of lactose and trehalose in lyophilized IPC-DNVs was a good strategy to obtain different sizes of IPC-DNVs (target size: 110 nm, 160 nm, 220 nm, 400 nm; actual size: 109.23 nm, 160.07 nm, 216.37 nm, 389.93 nm, respectively), while the EE% remained unaffected. Besides, when the content of sodium deoxycholate in IPC-DNVs changed from 0.5% to 1%, the particle size, size distribution, and EE% of DNVs did not change. However, deformability changed from 36.84 µg/cm^2^/s to 53.93 µg/cm^2^/s. The characterization results indicated that the IPC-DNVs, designed with different characteristics based on IPC-DNVs and lyophilized IPC-DNVs, met the quality requirements for buccal drug delivery.

In our previous study, the interaction between insulin and components in IPC-DNVs and lyophilized IPC-DNVs has been studied. The results showed that the components of IPC-DNVs had changed the insulin into amorphous form, but had no significant effect on the secondary structure of insulin, and also did not change the hypoglycemic properties and pharmacokinetics of insulin after subcutaneous administration. Considering that the ingredients of all formulation in this manuscript are consistent with IPC-DNVs and lyophilized IPC-DNVs, there is no further study on interaction of the insulin and components of those formulations in this manuscript.

### 4.3. Drug release *in vitro*

**Figure 1.**
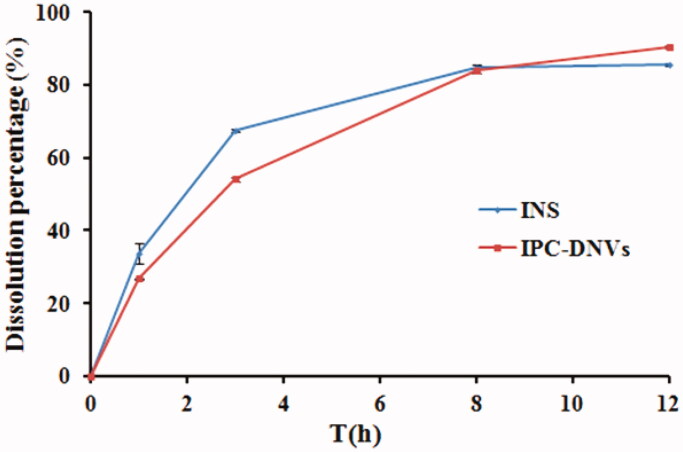
Drug release profile of IPC-DNVs and insulin (mean ± SD, *n* = 3).

The drug release profiles of insulin solution and IPC-DNVs were assessed in phosphate buffer (pH 7.4). The drug release profiles (Figure 1) indicated that insulin can be released rapidly from IPC-DNVs, the accumulated release amount of IPC-DNVs was >85% within 12 h, which was consistent with the release profiles of insulin solution. Combined with our previously study about the pharmacokinetics and *in vivo* hypoglycemic effect of IPC-DNVs and insulin solution after subcutaneous administration, insulin can release from the nanovesicles quickly, and the IPC-DNVs and insulin solution have the same pharmacokinetics characteristics and in *vivo* hypoglycemic properties, it can be concluded that the components and preparation process of IPC-DNVs had no significant effect on the release behavior of insulin *in vivo* and *in vitro* after insulin was made into IPC-DNVs.

### 4.4. *In vivo* hypoglycemic effect of dose

**Figure 2.**
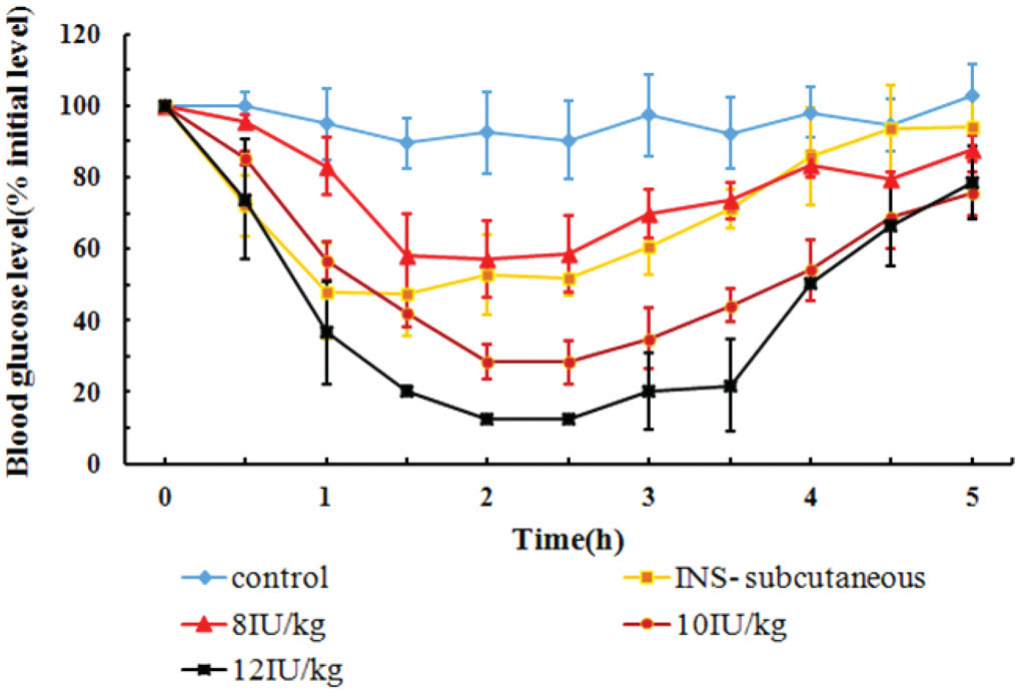
*In vivo* hypoglycemic effect of IPC-DNVs with different drug doses (mean ± SD, *n* = 3).

The hypoglycemic effects of IPC-DNVs, with different doses (8 IU/kg, 10 IU/kg, and 12 IU/kg), after buccal administration are shown in Figure 2. The glucose levels barely changed within 6 h after buccal administration of insulin solution, whereas hypoglycemic effect was evident in the INS-subcutaneous group and IPC-DNVs with different dose buccal administration group, thus suggesting that IPC-DNVs had good hypoglycemic effect. Unlike the dose of 8 IU/kg, which showed slight decrease (maximal decrease in blood glucose reached 58%) in blood glucose level, IPC-DNVs at a dose of 10 IU/kg and 12 IU/kg produced a significant hypoglycemic effect, with the maximal decrease in blood glucose reaching 30% and 12%, respectively, after buccal administration. Based on the above results, we concluded that the drug dose of IPC-DNVs had a significant effect on the hypoglycemic effect after buccal absorption. The increase in hypoglycemic effect in the first 2 h, when the insulin dose was increased from 8 to 12 IU/kg, could be attributed to the faster absorption of insulin from the oral mucosa due to the increased number of DNVs that reached the blood vessels for absorption (Sarmento et al., 2007). Table 1 summarizes the AAC_0–5h_ and Fp values of all the doses. A significant difference was observed in the respective AAC_0–5h_ and Fp values of doses 8 IU/kg and 10 IU/kg or 12 IU/kg, suggesting a non-liner dose response relationship in the range of 8–12 IU/kg. Interestingly, a relative lower Fp was observed at a dose of 8 IU/kg, which may be related to the mechanism of self-regulating glucose balance in normal animals (Benzo, 1983). When the dose of insulin is relatively low, normal rabbits can resist the excessive decrease in blood glucose through self-regulation. This explained the low Fp values for 8 IU/kg compared to that for 10/kg and 12 IU/kg. Since reduced glucose in plasma may be fatal to rabbits by causing hypoglycemia, amongst the three doses of IPC-DNVs, 10 IU/kg was found to be most adaptable dose in rabbits for buccal administration.

**Table 1.** Effect of different drug doses on AAC**_0–6h_** and Fp of IPC-DNVs (mean ± SD, *n* = 3).

Dose (IU/Kg)	AAC_0–5h_ (%·h)	Fp (%)
8	123.56 ± 33.43*^#^	9.68
10	255.19 ± 28.12	15.62
12	298.17 ± 35.51	15.57

*p < .05 between 8 IU and 10 IU, ^#^*p* < .01 between 8 IU and 12 IU.

### 4.5. *In vivo* hypoglycemic effect of drug delivery site

**Figure 3.**
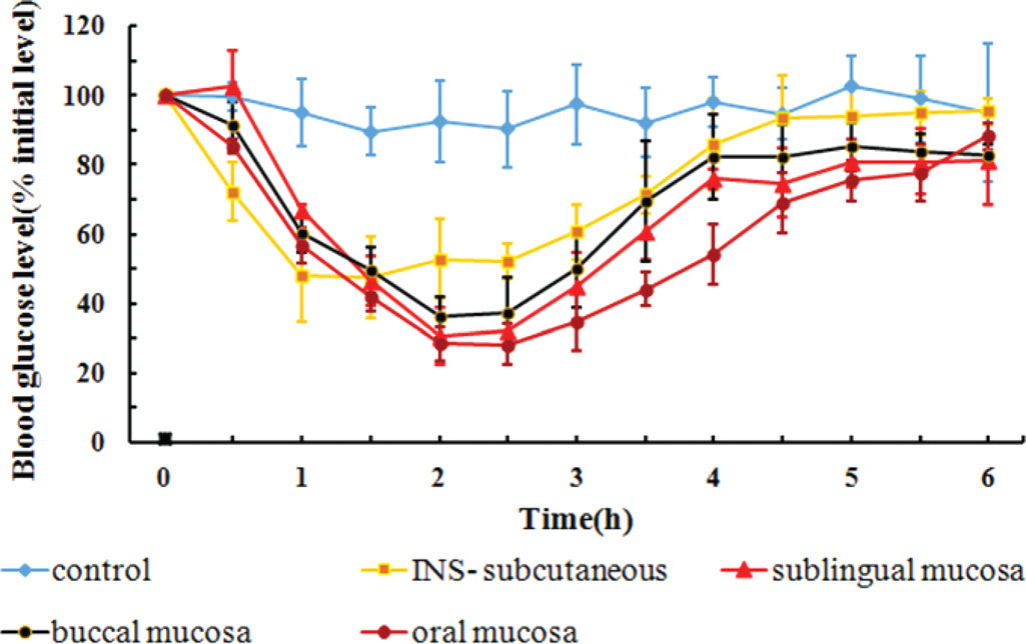
*In vivo* hypoglycemic effect of IPC-DNVs with different delivery sites (mean ± SD, *n* = 3).

The hypoglycemic effects after sublingual mucosal, buccal mucosal, and oral mucosal administration of IPC-DNVs are shown in Figure 3. The AAC_0–6h_ and Fp values are shown in Table 2. The hypoglycemic effects of sublingual and buccal mucosa administration of IPC-DNVs showed a similar trend, and there was no significant difference in their respective AAC_0–6h_ and Fp values. Sublingual and buccal mucosa have become the key routes for drug delivery due to their non-keratinized nature. However, different oral sites with the same pattern of epithelial differentiation (non-keratinization) do not necessarily exhibit the same permeability properties (Lesch et al., 1989). The results from this study showed that a similar hypoglycemic effect was obtained when the drug was administered via sublingual or buccal mucosa, which may be related to the larger surface area and higher degree of vascularization of the buccal mucosa (Patel et al., 2011). A significant difference in the AAC_0–6h_ between oral mucosa and buccal mucosa was observed, suggesting that for solid preparations or adhesive preparations with fixed area, sublingual mucosa administration might be a promising approach. For liquid preparations, however, oral mucosa administration (e.g. spray) remains the ideal mode for buccal delivery.

**Table 2.** Effect of different delivery sites on AAC**_0–6h_** and Fp of IPC-DNVs (mean ± SD, *n* = 3).

Administration site	AAC_0–6h_ (%·h)	Fp (%)
Sublingual mucosa	206.49 ± 37.99	12.64
Buccal mucosa	190.56 ± 45.93*	11.67
Oral mucosa	255.19 ± 28.12	15.62

*p < .05 between oral mucosa and buccal mucosa.

### 4.6. *In vivo* hypoglycemic effect of ligation time of IPC-DNVs

**Figure 4.**
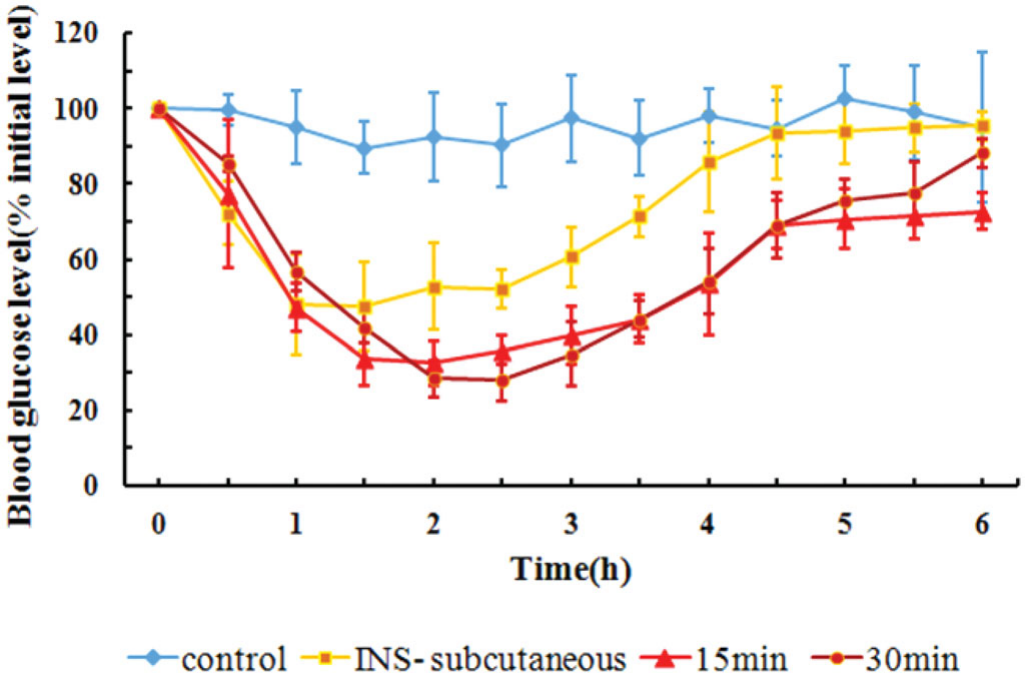
*In vivo* hypoglycemic effect of IPC-DNVs with different ligation times (mean ± SD, *n* = 3).

Drugs administered through buccal delivery cannot stay at the administration site for a long time due to mechanical stress and the continuous secretion of saliva (Hao & Heng, 2003). Thus, the rabbit esophageal ligation model was used in this study. We chose ligation times of 15 min and 30 min, which are commonly used in animal models. The hypoglycemic effects of ligation for 15 min or 30 min on rabbits are shown in Figure 4. The corresponding AAC_0–6h_ and Fp values are shown in Table 3. Contrary to the conventional understanding that prolonged ligation time might contribute to increased absorption of the drug, our study revealed less significant effects of ligation time on the buccal penetration of insulin, thus suggesting that the IPC-DNVs might be released and absorbed quickly in 15 min. Taking into consideration the esophagus operation time for ligation and untying, frequent surgery might make the animals intolerant. Hence, we chose 30 min as the ligation time in our study. However, when buccal administration is applied to the human body, secretion of saliva overtime results in increased fluid in the mouth, and thus makes it difficult for the patient to stop swallowing even for 15 min. To accurately predict the drug absorption effectiveness in the human body through the esophageal ligation model, it might be necessary to combine results on the absorption rate with the results of *in vivo* pharmacokinetics or pharmacodynamics studies.

**Table 3.** Effect of different ligation times on AAC**_0–6h_** and Fp of IPC-DNVs (mean ± SD, *n* = 3).

Ligation time (min)	AAC_0–6h_ (%·h)	Fp (%)
15	255.19 ± 28.12	15.62
30	268.97 ± 26.05	16.47

### 4.7. *In vivo* hypoglycemic effect of drug concentration of IPC-DNVs

**Figure 5.**
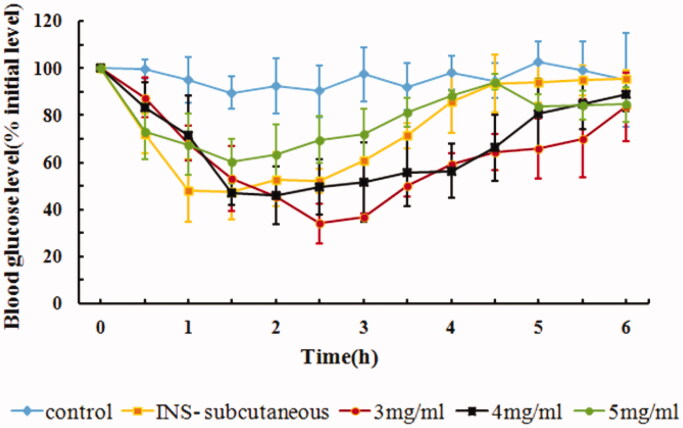
*In vivo* hypoglycemic effect of IPC-DNVs with different drug concentrations (mean ± SD, *n* = 3).

The administration volume of IPC-DNVs is limited due to the limited volume of the oral cavity. An increase in the volume of IPC-DNVs may increase the foreign body sensation in the mouth, thus promoting the secretion of saliva and inducing swallowing. Therefore, it is necessary to study the influence of lowering the administration volume. One of the methods for reducing administration volume is to increase the drug concentration of IPC-DNVs. The AAC_0–6h_ and Fp values of IPC-DNVs with different drug concentrations are shown in Table 4. As shown in Figure 5, it was found that by increasing the drug concentration from 3 mg/mL to 5 mg/mL, there was a decrease in AAC_0–6h_ and Fp of IPC-DNVs. This observation suggests that the insulin concentration of IPC-DNVs may be an important factor affecting the buccal delivery ability of the drug when the quality characteristics of IPC-DNVs are similar. It has been reported that as the concentration of deformable nanovesicles was increased, the cumulative drug transport improved, thus indicating a passive transport mechanism across the Caco-2 monolayers (Niu et al., 2014). However, in this study, as the insulin concentration increased, the AAC_0–6h_ decreased; suggesting that a passive transport mechanism may not exist for IPC-DNVs. According to our previous research, insulin–phospholipid complex might be mainly mediated by intercellular transport and DNVs might be mediated by paracellular transport (Xu et al., 2018). Another study also found that a large amount of insulin can deposit in the membrane after buccal administration of DNVs (Yang et al., 2002). Therefore, IPC-DNVs with higher insulin concentration might facilitate local leakage and accumulation of insulin during the squeezing of DNVs through the biological barriers, which in turn might decrease insulin absorption by hindering the continuous delivery of IPC-DNVs in the oral mucous membrane.

**Table 4.** Effect of different drug concentrations on size, PDI, EE, AAC**_0–6h_** and Fp of IPC-DNVs (mean ± SD, *n* = 3).

IPC-DNVs (drug concentration)	Size (nm)	PDI	EE (%)	AAC_0–6h_ (%·h)	Fp (%)
3mg/mL	109.23 ± 0.76	0.200 ± 0.009	80.50 ± 0.36	236.61 ± 37.94	14.49
4mg/mL	121.53 ± 2.16	0.267 ± 0.007	78.40 ± 0.78	208.49 ± 35.07	12.77
5mg/mL	113.97 ± 2.14	0.214 ± 0.011	79.46 ± 1.67	140.44 ± 34.11*	8.80

*p < .05 between 3 mg/mL and 5 mg/mL.

In our initial design of IPC-DNVs with different drug concentrations, we also attempted to study the influence of IPC-DNVs at a lower drug concentration. However, we found that due to the limitation of the volume of rabbit’s oral cavity, one of the three rabbits experienced drug overflow over the course of drug administration. These results suggest that the administration volume should not exceed the acceptable oral capacity, when administering DNVs via the buccal route.

### 4.8. *In vivo* hypoglycemic effect of size and deformability of IPC-DNVs

The size and deformability of DNVs are the key factors which affect their permeability. Yang and colleagues had reported that DNVs with a size of 42.5 nm exhibit good buccal absorption (Yang et al., 2002), while in our previous study, DNVs with a size of 80–110 nm showed a good hypoglycemic effect after buccal delivery (Xu et al., 2018). However, the upper size limit of DNVs that are able to cross the mucosal membranes remains unclear. While deformability, the main characterization parameter of DNVs, is only used in the prescription design of DNVs (Xu et al., 2019, Yusuf et al., 2014, Pathak et al., 2016), the effects of DNVs with different deformability *in vivo* has not yet been reported. According to a study, the intercellular space of oral mucosa is no more than 20 nm (Potts & Francoeur, 1990), and the maximum diameter that DNVs can penetrate is 10 times smaller than their own diameter (Rajan et al., 2011). Thus, the maximum diameter of DNVs should be less than 200 nm in theory. Based on this assumption, we designed IPC-DNVs with different target sizes (110 nm, 160 nm, 220 nm, and 400 nm). In addition, a surfactant with the action of edge activator was added to the phospholipid bilayer of the nanovesicles, which could destroy the balance of the phospholipid bilayer structure and make it deformable (Cevc & Blume, 1992). We thus designed IPC-DNVs with different deformabilities, ranging from 36.84 µg/cm^2^/s to 53.93 µg/cm^2^/s, by changing SDC concentrations to 0.5% and 1%, respectively.

**Figure 6.**
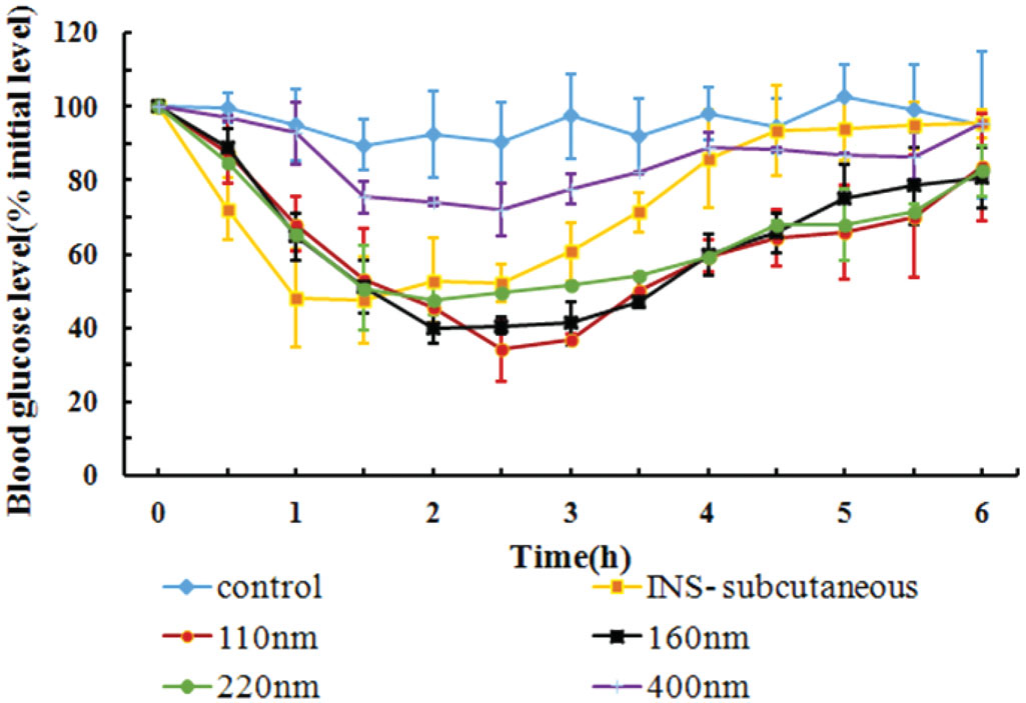
*In vivo* hypoglycemic effect of IPC-DNVs with different sizes (mean ± SD, *n* = 3).

The physical properties, AAC_0–6h_, Fp, and the hypoglycemic effects after buccal administration of IPC-DNVs of different sizes are shown in Table 5 and Figure 6. The Fp of IPC-DNVs of sizes 110 nm, 160 nm, and 220 nm had no obvious difference (14.49%, 14.01%, and 13.42%, respectively), and were similar to the Fp of IPC-DNVs 80 nm (15.62%). This indicated that for DNVs up to 200 nm, size variation may not affect buccal absorption significantly. However, there was a significant difference in Fp when the size was increased to 400 nm (5.52%). Combined with fact that the intercellular space of oral mucosa is not more than 20 nm, the better buccal absorption within the size range of 80 nm–220 nm can be explained by the ability of DNVs to penetrate membranes ten times their own diameter. It was also confirmed that when the size is beyond the deformation range, the delivery capacity of DNVs is significantly reduced. These results suggest that the size should be controlled within a reasonable range due to the limited deformability of DNVs.

**Table 5.** Effect of different sizes on PDI, EE, AAC**_0–6h_** and Fp of IPC-DNVs (mean ± SD, *n* = 3).

IPC-DNVs (size)	Size (nm)	PDI	EE (%)	AAC_0–6h_ (%·h)	Fp (%)
110nm	109.23 ± 0.76	0.200 ± 0.090	80.50 ± 0.36	236.61 ± 37.94	14.49
160nm	160.07 ± 3.55	0.325 ± 0.031	75.56 ± 1.37	228.88 ± 14.36	14.01
220nm	216.37 ± 2.29	0.427 ± 0.024	77.68 ± 0.96	219.19 ± 2.39	13.42
400nm	389.93 ± 8.39	0.564 ± 0.007	79.05 ± 1.02	90.00 ± 2.74**	5.52

**p < .01 between 400 nm and 110 nm or 160 nm or 220 nm.

**Figure 7.**
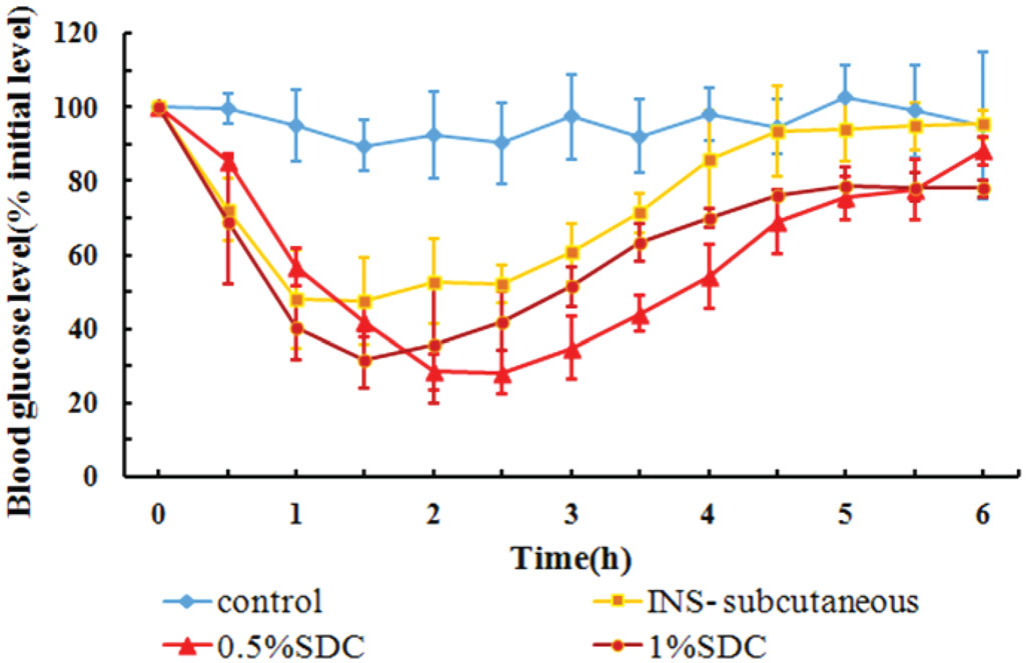
*In vivo* hypoglycemic effect of IPC-DNVs with different deformabilities (mean ± SD, *n* = 3).

Table 6 summarizes the physical properties, AAC_0–6h_, and Fp of IPC-DNVs with different deformabilities. The hypoglycemic effects after buccal administration of IPC-DNVs with different deformabilities are shown in Figure 7. The results suggested that IPC-DNVs with 0.5% SDC and 1% SDC had a significant hypoglycemic effect in rabbit, with no difference in their respective AAC_0–6h_ and Fp values.

**Table 6.** Effect of different concentrations of SDC (deformability) on size, PDI, EE, deformability, AAC**_0–6h_** and Fp of IPC-DNVs (mean ± SD, *n* = 3).

IPC-DNVs (amount of SDC)	Size (nm)	PDI	EE (%)	DI (µg/cm^2^/s)	AAC_0–6h_ (%·h)	Fp (%)
0.5%	81.41 ± 1.58	0.256 ± 0.004	73.23 ± 0.23	36.84 ± 1.47	255.19 ± 28.12	15.62
1%	73.70 ± 0.86	0.188 ± 0.006	77.40 ± 3.14	53.94 ± 3.04	237.26 ± 12.34	14.53

Our previous study had demonstrated that conventional nanovesicles without SDC, based on IPC-NVs, have little hypoglycemic effect. This indicates that DNVs made by adding SDC to conventional nanovesicles not only gives them deformability, but also significantly improves their mucosal permeability. However, it was reported that high deformability increases the leakage rate as vesicles lose some of their content during deformation while crossing the pores (Kumar et al., 2012). Furthermore, certain drugs such as phospholipid complex may have their own absorption ability. It is, therefore, difficult to predict the degree of absorption *in vivo* because of the uncertainty regarding the amount of drug leaked due to high deformability and the absorption degree of the leaked drug itself in the mucous membrane. Thus, to obtain a suitable deformability, it is necessary to consider not only the storage stability that might be caused by high deformability, but also the effective delivery ability of DNVs *in vivo*, combined with pharmacokinetics or pharmacodynamic experiments. In this study, we found that the blood glucose levels decreased faster within 1.5 h, when IPC-DNVs with higher deformability were used, which may be related to the faster penetration time of IPC-DNVs with high deformability. However, further increase in the deformability and IPC deposition of the initial IPC-DNVs did not improve buccal absorption.

## 5. Conclusion

*In vivo* investigations were conducted to elucidate the factors affecting the buccal delivery of IPC-DNVs. In this study, we first studied the stability of IPC-DNVs on degradation by buccal enzymes. Next, we systematically studied the influence of drug dose, buccal drug administration method (delivery site, ligation time), and the key quality characteristics of IPC-DNVs (drug concentration, size, and deformability) on buccal delivery. We found that IPC-DNVs can be fully absorbed in 15 min and that they showed a non-linear dose–response relationship between insulin dose of 8 IU/kg and 12 IU/kg. The delivery site and ligation time had no influence on buccal absorption. However, the AAC_0–6h_ of oral mucosal administration was significantly higher than that of the buccal mucosal administration, suggesting that oral mucosal administration is more suitable for the buccal delivery of DNVs. Moreover, increased drug concentration led to decrease in AAC_0–6h_ and Fp. This may be due to the local leakage and accumulation of IPC-DNVs, with high concentration of insulin squeezing through the biological barrier hindering the delivery of subsequent IPC-DNVs. In addition, in terms of two key characteristics of IPC-DNVs, increasing size beyond a certain range (80 nm–220 nm) had no significant effect on the oral absorption. However, when the size was increased to 400 nm, the AAC_0–6h_ decreased significantly, indicating that in the prescription design of DNVs, the size should be controlled within a reasonable. Further, increasing deformability above the existing deformability range was found to be ineffective in promoting absorption. It is also necessary to optimize the deformability, combined with *in vivo* efficacy for better drug delivery through the buccal route. Our results not only clarified the characteristics of IPC-DNVs on buccal delivery, but also provide meaningful evidence for dosage form design and administration methods for buccal delivery system of IPC-DNVs.
